# Nicht-Cholera-Vibrionen – derzeit noch seltene, aber wachsende Infektionsgefahr in Nord- und Ostsee

**DOI:** 10.1007/s00108-021-01086-x

**Published:** 2021-07-16

**Authors:** Thomas Theo Brehm, Susann Dupke, Gerhard Hauk, Helmut Fickenscher, Holger Rohde, Laura Berneking

**Affiliations:** 1 I. Medizinische Klinik und Poliklinik, Sektion Infektiologie, Universitätsklinikum Hamburg-Eppendorf, Martinistr. 52, 20246 Hamburg, Deutschland; 2 Deutsches Zentrum für Infektionsforschung (DZIF), Standort Hamburg-Lübeck-Borstel-Riems, http://www.dzif.de; 3 Robert Koch Institut, ZBS 2: Hochpathogene mikrobielle Erreger, Berlin, Deutschland; 4 Landesamt für Gesundheit und Soziales Mecklenburg-Vorpommern, Abteilung Gesundheit, Dezernat Umwelthygiene, Umweltmedizin, Rostock, Deutschland; 5 Institut für Infektionsmedizin, Christian-Albrechts-Universität zu Kiel und Universitätsklinikum Schleswig-Holstein, Kiel, Deutschland; 6 Institut für Medizinische Mikrobiologie, Virologie und Hygiene, Universitätsklinikum Hamburg-Eppendorf, Hamburg, Deutschland

**Keywords:** Deutschland, Klimawandel, Meerwasser, Wasserbezogene Krankheit, Wundinfektionen, Germany, Climate change, Seawater, Waterborne diseases, Wound infection

## Abstract

**Hintergrund:**

Nicht-Cholera-Vibrionen nehmen im Rahmen des Klimawandels eine zunehmende Bedeutung als humane Pathogene ein, da die Prävalenz dieser Erreger im Meereswasser entscheidend von der Wassertemperatur abhängt. In den letzten Jahren konnten während der Sommermonate wiederholt größere Infektionsausbrüche in gemäßigten Klimazonen beobachtet werden.

**Ziel der Arbeit:**

Information einer breiten ärztlichen Leserschaft über potenziell lebensbedrohliche Krankheitsbilder, die durch Infektionen mit Nicht-Cholera-Vibrionen ausgelöst werden.

**Material und Methoden:**

Übersicht über aktuelle Literatur zu Infektionen mit Nicht-Cholera-Vibrionen im Allgemeinen und zur epidemiologischen Situation in Deutschland im Speziellen.

**Ergebnisse:**

Nicht-Cholera-Vibrionen verursachen vorwiegend Wund- und Ohrinfektionen nach Kontakt mit kontaminiertem Meereswasser sowie Gastroenteritiden nach dem Konsum nicht ausreichend gegarter Meerestiere. Da bis März 2020 keine Meldepflicht für diese Erreger in Deutschland bestand, muss von einer hohen Dunkelziffer ausgegangen werden. Immunsupprimierte sowie chronisch erkrankte Menschen haben ein deutlich erhöhtes Risiko für schwere Krankheitsverläufe. Schon bei klinischem Verdacht sollte eine kalkulierte antiinfektive Therapie erfolgen und bei Wundinfektionen eine chirurgische Sanierung erwogen werden.

**Diskussion:**

Aufgrund des fortschreitenden Klimawandels muss in den kommenden Jahren mit dem vermehrten Auftreten von Infektionen mit Nicht-Cholera-Vibrionen gerechnet werden. Ärzte sollten über diese potenziell lebensbedrohlichen Erkrankungen informiert sein, um Patienten einer entsprechenden Diagnostik und Behandlung zuzuführen.

## Abstract (en)

**Background:**

The abundance of non-cholera *Vibrio* spp. in the aquatic environment shows a positive correlation with water temperatures. Therefore, climate change has an important impact on the epidemiology of human infections with these pathogens. In recent years large outbreaks have been repeatedly observed during the summer months in temperate climate zones.

**Objective:**

To inform medical professionals about the potentially life-threatening diseases caused by non-cholera *Vibrio* spp.

**Material and methods:**

Review of the current literature on infections with non-cholera *Vibrio* spp. in general and on the epidemiological situation in Germany in particular.

**Results:**

Non-cholera *Vibrio* spp. predominantly cause wound and ear infections after contact with contaminated seawater and gastroenteritis after consumption of undercooked seafood. As there have not been mandatory notification systems for these pathogens in Germany up to March 2020, a high number of unreported cases must be assumed. Immunosuppressed and chronically ill patients have a much higher risk for severe courses of diseases. If an infection with non-cholera *Vibrio* spp. is suspected anti-infective treatment should be promptly initiated and surgical cleansing is often necessary for wound and soft tissue infections.

**Conclusion:**

Due to the ongoing global warming an increased incidence of human infections with non-cholera *Vibrio* spp. must be expected in the future. Medical professionals should be aware of these bacterial pathogens and the potentially life-threatening infections in order to enable timely diagnostics and treatment.

Die globale Erwärmung stellt die wohl größte Herausforderung des 21. Jahrhunderts dar [20]. In den kommenden Jahrzehnten ist weltweit mit einem erheblichen Anstieg der vom Klimawandel verursachten Morbidität und Mortalität zu rechnen, wobei neben Naturkatastrophen, Luftverschmutzung und Mangelernährung auch zahlreiche Infektionskrankheiten von Bedeutung sind [16, 18]. Einen wichtigen Stellenwert nehmen Nicht-Cholera-Vibrionen ein, die in salzhaltigen Gewässern vorkommen und zunehmend teils schwer verlaufende Infektionen verursachen [3].

## Mikrobiologie

Vibrionen sind leicht gekrümmte gramnegative Stäbchen mit meist unipolarer Geißel aus der Familie der *Vibrionaceae*, die eine Vielzahl verschiedener Spezies umfasst. Toxinbildende *V.-cholerae-*Stämme der Serogruppen O1 und O139 sind die Auslöser der Cholera, die heute ausschließlich in Ländern mit Mangel an sauberem Trinkwasser durch unzureichende Sanitäranlagen vorkommt [10]. Andere Serogruppen von *V. cholerae* sowie andere humanpathogene Spezies der Gattung *Vibrio* werden auch als Nicht-Cholera-Vibrionen bezeichnet. Diese werden im Gegensatz zu choleratoxinbildenden *Vibrio*-Spezies nicht von Mensch zu Mensch übertragen und lösen daher keine Epidemien aus. Sie sind weltweit Bestandteil der bakteriellen Flora schwach salzhaltiger Gewässer in Küstennähe, wie Flussmündungen, Buchten, Bodden und Brackwässer [3], und kommen auch in Binnenseen wie dem Neusiedler See in Österreich vor [32]. Nicht-Cholera-Vibrionen treten sowohl frei im Wasser auf, können aber auch als Kommensalen oder als Pathogene von Meerestieren fungieren. Insgesamt wurden von den weit über 100 bekannten Spezies bislang 13 als humanpathogen beschrieben (Tab. 1; [11, 21]).

**Table 1:** 

*Vibrio*-Spezies	Manifestation	
	Gastrointestinal	Extraintestinal
*V. alginolyticus*	–	++
*V. cholerae*		
– O1/O139	++++	+
– Non-O1/O139	++	++
*V. cincinnatiensis*	–	+
*V. fluvialis*	++	–
*V. furnissii*	++	–
*V. harveyi*	–	+
*V. hollisae*	++	–
*V. navarrensis*	–	+
*V. metschnikovii*	+	+
*V. mimicus*	++	+
*V. parahaemolyticus*	++++	+
*V. vulnificus*	+	+++

## Prävalenz von *Vibrio*-Spezies in Nord- und Ostsee

Die Prävalenz von Vibrionen und das daraus resultierende Infektionsrisiko hängen im Wesentlichen von der Temperatur sowie vom Salzgehalt des Wassers ab [3]. Während in den Tropen ganzjährlich Infektionen mit Nicht-Cholera-Vibrionen auftreten, weisen diese in den gemäßigten Klimazonen eine stark ausgeprägte Saisonalität auf [5]. Die überwiegende Mehrzahl der Fälle kommt hier in den warmen Sommermonaten vor, wenn die Temperatur des Oberflächenwassers 20 °C übersteigt [7]. Zwar lassen sich auch bei niedrigeren Wassertemperaturen regelmäßig Vibrionen in Nord- und Ostsee nachweisen, jedoch führt die dann meist niedrige Bakterienlast nur sehr selten zu humanen Infektionen [7].

Ansteigende Oberflächentemperatur und sinkender Salzgehalt können die Verbreitung von Vibrionen im Meer begünstigen

**Figure 1:**
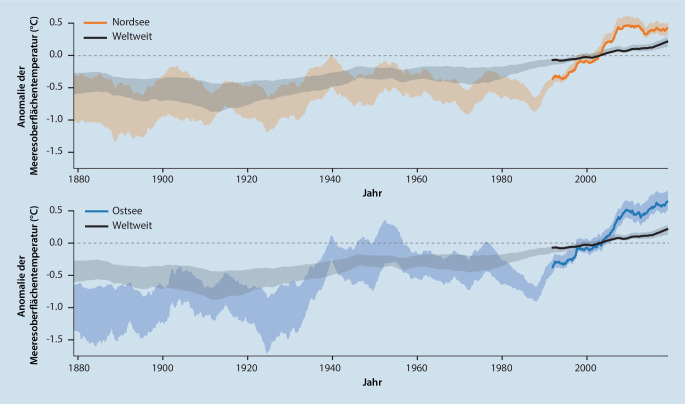

Bereits heute lässt sich in allen Weltmeeren ein durch den Klimawandel bedingter Anstieg der Oberflächentemperatur verzeichnen ([1]; Abb. 1). Im Rahmen sommerlicher Hitzewellen wurden in den letzten Jahren vielerorts noch nie dagewesene Spitzenwerte der Wassertemperaturen verzeichnet [3]. Besonders ausgeprägt ist diese Entwicklung in der Ostsee, die weltweit eines der Meeresökosysteme darstellt, die sich am schnellsten erwärmen [19]. Hier wird in den kommenden Jahrzehnten ein weiterer Anstieg der Oberflächentemperatur um voraussichtlich 4–5 °C erwartet, was mit einer deutlich erhöhten Dichte an Vibrionen einhergehen wird [33]. Neben der Wassertemperatur spielt der Meeressalzgehalt eine wesentliche Rolle für das Wachstum von Vibrionen. Diese sind moderat halophil, vermehren sich also bevorzugt bei eher niedrigem Salzgehalt. Während die Ostsee mit einem durchschnittlichen Salzgehalt von 0,8 % optimale Bedingungen bietet, ist die offene Nordsee mit einem Salzgehalt von 3,5 % vielerorts für eine effiziente Vermehrung der meisten *Vibrio*-Spezies nicht geeignet [24]. Allerdings sind im Bereich von Flussmündungen häufig niedrigere Salinitäten zu finden. In den nächsten Jahren könnte es zu einer weiteren Abnahme des Salzgehaltes von Nord- und Ostsee kommen und somit die Verbreitung von Vibrionen in Deutschland weiter begünstigt werden [15, 27].

## Klinische Manifestationen

Nicht-Cholera-Vibrionen können sowohl gastrointestinale Symptome, Wund- und Ohrinfektionen als auch schwerste septische Verläufe verursachen, wobei sich die einzelnen Spezies in ihrer jeweiligen Virulenz unterscheiden [2, 11]. Insbesondere Infektionen mit *V. vulnificus* sind häufig foudroyant und zeichnen sich durch eine hohe Mortalität bis zu 50 % aus [29]. Gastrointestinale Infektionen treten überwiegend nach dem Konsum roher oder nicht ausreichend gegarter Meerestiere (z. B. Muscheln, Austern, Krabben) auf; der klinische Verlauf ist bei Immunkompetenten in der Mehrzahl der Fälle eher mild. Eintrittspforten für durch Nicht-Cholera-Vibrionen ausgelöste Wundinfektionen sind meist Hautwunden oder eine chronisch geschädigte Hautbarriere.

Infektionen mit *V. vulnificus* zeichnen sich durch eine besonders hohe Mortalität aus

**Figure 2:**
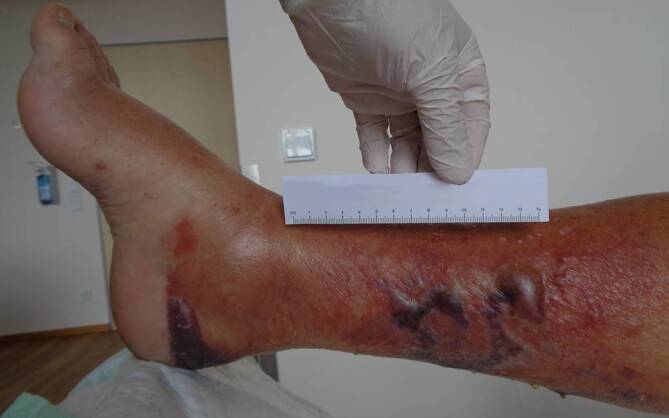

Die Übertragung kann beim Baden in erregerhaltigem Wasser oder bei der Verarbeitung kontaminierter Meerestiere erfolgen. Solche Wundinfektionen können mit Blasenbildung einhergehen, sich rasch ausbreiten und zu tiefgreifenden Hautulzerationen und Nekrosen führen, die in schweren Fällen eine Amputation erforderlich machen (Abb. 2). Bei prädisponierten Personen kann sich ferner eine fulminante Sepsis mit Multiorganversagen entwickeln. Ein erhöhtes Risiko für schwere Krankheitsverläufe weisen ältere Menschen, immunsupprimierte Patienten sowie Patienten mit chronischen Erkrankungen wie chronischer Niereninsuffizienz, schweren Herz-Kreislauf-Erkrankungen oder Diabetes mellitus, insbesondere bei schlechter Stoffwechseleinstellung, auf. Diabetische Fußsyndrome können zudem als potenzielle Eintrittspforten für Nicht-Cholera-Vibrionen dienen. Eine besonders hohe Letalität haben ferner Menschen mit fortgeschrittenen Lebererkrankungen [12]. Bei Patienten mit chronischen Hepatopathien im Allgemeinen und der Hämochromatose im Speziellen liegt regelhaft ein erhöhter Eisenspiegel vor; dies birgt ein erhöhtes Risiko für schwere Krankheitsverläufe, da viele Nicht-Cholera-Vibrionen für ihren Stoffwechsel auf Eisen angewiesen sind [2]. Auch Ohrinfektionen können nach dem Baden in mit Nicht-Cholera-Vibrionen kontaminiertem Wasser auftreten. Diese betreffen meist den äußeren Gehörgang und deutlich seltener das Mittelohr.

## Inzidenz humaner Infektionen

Aufgrund der globalen Erwärmung wurde in den letzten Jahren weltweit eine Zunahme von Erkrankungen, die durch Infektionen mit Nicht-Cholera-Vibrionen ausgelöst werden, verzeichnet [5]. Auch in gemäßigten Klimazonen wie Schweden und Finnland [4], Spanien [26], Israel [31], Peru [14] oder den USA [25] kam es in den Sommermonaten wiederholt zu größeren Ausbrüchen. In den USA, in denen im Gegensatz zu den meisten europäischen Ländern eine allgemeine Meldepflicht für Erkrankungen durch Nicht-Cholera-Vibrionen besteht und somit belastbare Fallzahlen vorliegen, verdreifachte sich die jährliche Inzidenz von 0,09/100.000 im Jahr 1996 auf 0,29/100.000 im Jahr 2010 [30].

## Infektionsfälle in Deutschland

**Figure 3:**
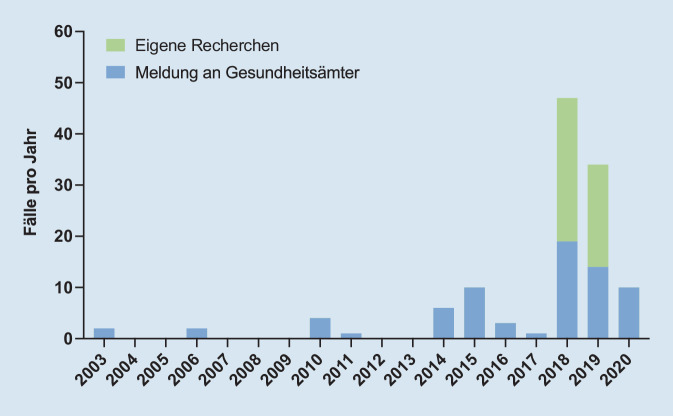

**Figure 4:**
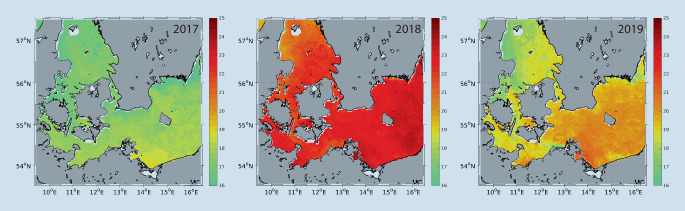

In Deutschland gab es gemäß dem Infektionsschutzgesetz (IfSG) bis vor Kurzem keine Meldepflicht für Infektionen mit Nicht-Cholera-Vibrionen, weshalb diese nicht systematisch erfasst und in ihrer Bedeutung sicherlich unterschätzt wurden. Der erste Fall einer in Deutschland erworbenen Infektion mit Nicht-Cholera-Vibrionen wurde 1994 bei einer 71-jährigen Urlauberin beschrieben. Diese erlitt einen septischen Schock mit Nachweis von *V. vulnificus*, nachdem sie zuvor auf Usedom mit einer kleinen Wunde am Bein durchs Meerwasser gewatet war [17]. Zwischen 2003 und 2017 wurden den Landesmeldestellen Schleswig-Holstein und Mecklenburg-Vorpommern nur wenige Erkrankungsfälle mit Nicht-Cholera-Vibrionen angezeigt, die in Form wöchentlicher Meldeberichte der Öffentlichkeit zu Verfügung gestellt werden. Die meisten Infektionen mit 10 bzw. 6 gemeldeten Fällen waren während der besonders heißen Sommer der Jahre 2014 und 2015 zu verzeichnen (Abb. 3). In den beiden Jahren 2018 und 2019 kam es zu außergewöhnlichen sommerlichen Hitzewellen (Abb. 4), während derer den Landesmeldestellen insgesamt 33 Infektionsfälle gemeldet wurden.

Wir führten darüber hinaus in den Jahren 2018 und 2019 gemeinsam mit dem Robert Koch-Institut, in dem das Speziallabor für hochpathogene mikrobielle Erreger angesiedelt ist, eine Befragung v. a. norddeutscher Kliniken und Labore zu klinischen Fällen aus den Jahren 2018 und 2019 durch und konnten zusätzliche 48 aufgetretene Infektionsfälle identifizieren [9]. Seit dem 01.03.2020 besteht gemäß IfSG eine namentliche Meldepflicht für alle Infektionen mit humanpathogenen Vibrionen. Labore müssen Nachweise von *Vibrio*-Spezies gemäß § 7 Abs. 1 namentlich melden, sofern der Nachweis auf eine akute Infektion hinweist. Lediglich im Fall einer ausschließlich das Ohr betreffenden Infektion gilt dies nur für *V. cholerae*. Die trotz eingeführter Meldepflicht im Vergleich zu den Vorjahren geringen Infektionszahlen im Jahr 2020 (*n* = 10) sind sicherlich auch auf den eingeschränkten Badebetrieb zurückzuführen; dieser war durch die Pandemie, die die „coronavirus disease 2019“ (COVID-19) verursacht hat, bedingt. Von den insgesamt 120 den Landesmeldestellen gemeldeten bzw. durch eigene Recherchen identifizierten Infektionen mit Nicht-Cholera-Vibrionen zwischen 2003 und 2020 wurden 118 an der Ostsee und 2 Infektionen an der Nordsee erworben.

**Figure 5:**
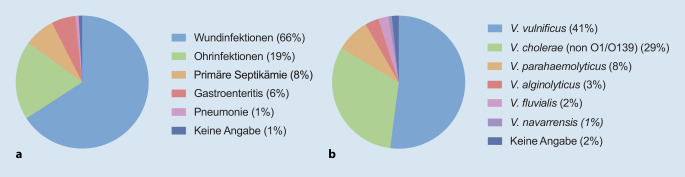

Den größten Anteil machten Wundinfektionen aus; seltener waren Ohrinfektionen, primäre Septikämien oder Gastroenteritiden (Abb. 5). Bei den betroffenen Patienten wurde am häufigsten *V. vulnificus* nachgewiesen, gefolgt von *V. cholerae *(non-O1/non-O139), *V. parahaemolyticus, V. alginolyticus, V. fluvialis und V. navarrensis.* Insgesamt 15 mehrheitlich männliche Patienten mit einem medianen Alter von 70 Jahren (Range 56 bis 93 Jahre) verstarben an ihrer Infektion (Tab. 2). Der Großteil dieser Patienten erlag an Krankheitsbildern, die durch eine Infektion mit *V. vulnificus* hervorgerufen worden waren. Lediglich 2 Patienten hatten sich mit *V. cholerae* (non-O1/non-O139) infiziert. Die Mehrheit der Patienten mit letalem Verlauf hatte sich beim Baden in der Ostsee Wundinfektionen zugezogen, häufig wurde auch eine Bakteriämie nachgewiesen. Andere Infektionswege waren der Konsum von Krabben bzw. Fischzubereitungen sowie die Aspiration von Meerwasser. Alle bis auf einen der verstorbenen Patienten litten an chronischen Erkrankungen; fünf dieser Patienten waren durch Medikamentenanwendung oder Grunderkrankungen immunsupprimiert. Setzt man die genannten Fallzahlen und Todesfälle ins Verhältnis zur Gesamtzahl potenziell exponierter Besucher an der deutschen Nord- und Ostseeküste – pro Jahr etwa 36 Mio. touristische Übernachtungen in der Region Ostsee und über 10 Mio. Übernachtungen in der Region Nordsee [22, 23] –, wird deutlich, dass durch Nicht-Cholera-Vibrionen-Infektionen ausgelöste Erkrankungen, selbst unter der Annahme einer relevanten Dunkelziffer, aktuell weiterhin eher seltene Ereignisse sind.

**Table 2:** 

Jahr	Alter (Jahre)	Geschlecht	*Vibrio*-Spezies	Klinische Manifestation	Chronische Erkrankung	Immunsuppression
2003	62	Männlich	*V. vulnificus*	Wundinfektion	Ja	Nein
2010	62	Weiblich	*V. cholerae*	Wundinfektion	Ja	Nein
2010	81	Weiblich	*V. vulnificus*	Wundinfektion mit Bakteriämie	Ja	Nein
2014	59	Weiblich	*V. vulnificus*	Wundinfektion	Ja	Nein
2018	69	Männlich	*V. vulnificus*	Wundinfektion mit Bakteriämie	Ja	Nein
2018	76	Männlich	*V. vulnificus*	Wundinfektion	Ja	Nein
2018	82	Männlich	*V. vulnificus*	Wundinfektion	Ja	Ja
2018	77	Männlich	*V. vulnificus*	Wundinfektion	Ja	Nein
2018	70	Männlich	*V. vulnificus*	Wundinfektion mit Bakteriämie	Ja	Ja
2018	63	Männlich	*V. vulnificus*	Sepsis nach Fischzubereitung und -konsum	Ja	Ja
2019	88	Männlich	*V. cholerae*	Sepsis nach Konsum von Krabben	Ja	Ja
2019	82	Männlich	*V. vulnificus*	Wundinfektion mit Bakteriämie	Ja	Nein
2019	68	Männlich	*V. vulnificus*	Sepsis nach Aspiration	Nein	Nein
2019	93	Weiblich	*V. vulnificus*	Wundinfektion mit Bakteriämie	Ja	Ja
2019	56	Männlich	*V. vulnificus*	Wundinfektion mit Bakteriämie	Ja	Nein

## Diagnostik

Nicht-Cholera-Vibrionen lassen sich aus Stuhl, Blutkulturen und Wundmaterial von Patienten auf Blutagar und selektiv auf „Thiosulfate-citrate-bile-sucrose“(TCBS)-Agar kultivieren. Die im Selektivagar enthaltene Saccharose erlaubt zusammen mit pH-Indikatoren eine Diskriminierung einzelner *Vibrio*-Spezies anhand der unterschiedlichen Färbungen der gewachsenen Kolonien [2]. Die weitere Speziesidentifizierung und Subtypisierung gewonnener Patientenisolate gelingt mithilfe klassischer mikrobiologischer Methoden auf biochemischer und immunologischer Basis oder dem molekulargenetischen Nachweis spezifischer Genmarker in Echtzeit („Real-time“-Polymerase-Kettenreaktion, PCR, [28]). Außerdem verfügen mittlerweile viele Labore über gängige Methoden zur Untersuchung mithilfe der Massenspektrometrie (z. B. Kombination der *m*atrix*a*ssistierten *L*aser-*D*esorption-*I*onisierung mit der „Time-of-flight“-Analyse, MALDI-TOF-MS; [13]).

## Therapie

Aufgrund der potenziellen Fulminanz einer Erkrankung, die durch eine Infektion mit Nicht-Cholera-Vibrionen ausgelöst wurde, ist bei entsprechendem Verdacht die frühestmögliche Einleitung der kalkulierten antibiotischen Therapie noch vor der mikrobiologischen Bestätigung der Infektion von entscheidender prognostischer Bedeutung. Meist werden Cephalosporine der 3. Generation, Tetrazykline oder Gyrasehemmer als Mono- oder Kombinationstherapie empfohlen [34, 35].

Die zunehmenden Antibiotikaresistenzen der Nicht-Cholera-Vibrionen sind besorgniserregend

Zwar sind Nicht-Cholera-Vibrionen empfindlich gegenüber den meisten bei gramnegativen Erregern wirksamen Antibiotikaklassen. Bei aus Nord- und Ostsee stammenden Nicht-Cholera-Vibrionen konnten jedoch bereits vermehrt Resistenzen gegenüber Aminoglykosiden, Aminopenicillinen und Streptomycin gezeigt werden [6]. Zudem wurden einzelne Isolate mit Carbapenem-hydrolysierenden β‑Lactamasen nachgewiesen. Diese zunehmenden Antibiotikaresistenzen bei Nicht-Cholera-Vibrionen sind in mehrfacher Hinsicht besorgniserregend. Zum einen kann eine nicht uneingeschränkt wirksame kalkulierte antibiotische Therapie bei fulminanter Krankheit verhängnisvoll sein. Zum anderen muss befürchtet werden, dass Nicht-Cholera-Vibrionen als Reservoir für die Übertragung plasmidkodierter Antibiotikaresistenzen fungieren könnten. Neben der Einleitung einer antibiotischen Therapie muss bei Wund- und Weichteilinfektionen immer eine frühzeitige chirurgische Sanierung in Erwägung gezogen werden; im fortgeschrittenem Krankheitsstadium ist eine Amputationen häufig unvermeidlich [12].

## Prävention

Da Infektionen mit Nicht-Cholera-Vibrionen bei immunsupprimierten oder chronisch kranken Menschen besonders häufig mit einem schweren Verlauf einhergehen, sollten diese Risikogruppen angehalten werden, insbesondere bei Vorliegen von Hautverletzungen, während heißer Sommermonate den Kontakt zu salzhaltigen Gewässern zu meiden. Urlaube an Nord- und Ostsee sind insbesondere bei Menschen mit chronischen Hauterkrankungen wie Psoriasis und atopischer Dermatitis weit verbreitet; diese sind beim Vorliegen offener Wunden jedoch einem erhöhten Risiko für Infektionen mit Nicht-Cholera-Vibrionen an Nord- und v. a. Ostsee ausgesetzt. Zur Prävention von Lebensmittelinfektionen sollten Meerestiere nicht roh oder unzureichend gegart verzehrt und bei der Zubereitung von Schalentieren Verletzungen der Haut vermieden werden. Regelmäßige Umweltuntersuchungen zur sommerlichen Bakterienlast in Nord- und Ostsee werden zwar durchgeführt, jedoch können in Ermangelung klarer Grenz- oder Maßnahmenwerte keine eindeutigen Handlungsempfehlungen abgeleitet werden [24].

Der „Vibrio Map Viewer“ errechnet das jeweils tagesaktuelle Risiko für Infektionen mit Vibrionen

Die Landesbehörden haben in der Vergangenheit nach bekannt gewordenen Infektionen jeweils sowohl an Ärzte als auch an die Bevölkerung gerichtete Informationsschreiben zur potenziellen Gefahr der Infektionen mit Nicht-Cholera-Vibrionen veröffentlicht. Als zusätzliche Informationsquelle stellt das European Centre for Disease Prevention and Control (ECDC) den „Vibrio Map Viewer“ zur Verfügung, eine online einsehbare interaktive Karte der Ostsee, die das jeweils tagesaktuelle Risiko für Infektionen mit Vibrionen aus den Oberflächentemperaturen der Ostsee und dem Salzgehalt errechnet [33].

## Resümee

Der Klimawandel wird in den kommenden Jahren zum deutlichen Anstieg der Oberflächentemperaturen von Nord- und Ostsee führen, weshalb ein gehäuftes Vorkommen von Nicht-Cholera-Vibrionen zu erwarten ist. Darüber hinaus wird der demografische Wandel eine Zunahme vulnerabler Bevölkerungsgruppen mit sich bringen. Die Inzidenz humaner Infektionen mit Nicht-Cholera-Vibrionen wird folglich voraussichtlich beträchtlich ansteigen. Während der warmen Sommermonate muss bei verdächtigem Krankheitsbild stets die Möglichkeit einer Infektion mit Nicht-Cholera-Vibrionen in Erwägung gezogen und der Kontakt mit salzhaltigem Wasser bzw. der Verzehr von möglicherweise unzureichend gegarten Meerestieren erfragt werden. Aufgrund des Binnentourismus an Nord- und Ostsee ist eine erhöhte Wachsamkeit auch der in anderen Landesteilen praktizierenden Ärzte geboten. Aber auch bei einem entsprechendem Krankheitsbild von Reiserückkehrern muss an Infektionen mit Nicht-Cholera-Vibrionen gedacht werden. Dies verdeutlicht der Fall eines 2018 im Universitätsklinikum Hamburg-Eppendorf behandelten Patienten, der sich auf Mallorca im Rahmen einer Schiffsschraubenverletzung mit Amputation des Unterschenkels eine Wundinfektion mit *V. harveyi* zugezogen hatte [8]. Um in Zukunft tiefergehende Erkenntnisse über Epidemiologie, Mikrobiologie und klinische Zeichen von Infektionen mit Nicht-Cholera-Vibrionen zu gewinnen, ist neben einer zuverlässigen Surveillance dringend auch weitere Forschung zu diesen neu aufkommenden Pathogenen erforderlich.

## Fazit für die Praxis

- Infektionen mit Nicht-Cholera-Vibrionen können während heißer Sommermonate in Nord- und Ostsee erworben werden.
- Aufgrund des Klimawandels sowie der Zunahme vulnerabler Bevölkerungsgruppen ist in Zukunft von einem vermehrten Auftreten von Infektionen mit Nicht-Cholera-Vibrionen auszugehen.
- Risikogruppen wie Menschen mit chronischen Erkrankungen (insbesondere fortgeschrittene Lebererkrankungen und Diabetes mellitus mit schlechter Stoffwechseleinstellung) sowie Immunsupprimierte sollten insbesondere bei Vorliegen von Hautverletzungen während heißer Sommermonate den Kontakt zu Salzwasser aus Nord- und Ostsee meiden.
- Ärzte in ganz Deutschland sollten über Infektionen mit Nicht-Cholera-Vibrionen und deren potenzielle Fulminanz informiert sein und Patienten mit verdächtigen Krankheitsbildern (Wundinfektionen, primären Septikämien, Gastroenteritis, Ohrinfektionen) gezielt nach entsprechender Exposition befragen.
- Bei begründetem Verdacht auf eine Infektion mit Nicht-Cholera-Vibrionen sollte unverzüglich mit einer kalkulierten antibiotischen Therapie begonnen sowie bei Wund- und Weichteilinfektionen eine chirurgische Sanierung in Erwägung gezogen werden.
